# Molecular Dynamics Simulations on the Elastic Properties of Polypropylene Bionanocomposite Reinforced with Cellulose Nanofibrils

**DOI:** 10.3390/nano12193379

**Published:** 2022-09-27

**Authors:** Vaibhav Modi, Antti J. Karttunen

**Affiliations:** Department of Chemistry and Materials Science, Aalto University, FI-00076 Aalto, Finland

**Keywords:** bionanocomposites, nanocellulose, polypropylene, maleic anhydride, molecular dynamics, elastic properties

## Abstract

Cellulose-reinforced polypropylene bionanocomposites can show improved elastic properties over their pure polypropylene counterparts. We have used equilibrium and non-equilibrium molecular dynamics (MD) simulations to study the elastic properties of polypropylene bionanocomposite systems composed of cellulose nanofibrils (CNF), polypropylene (PP) matrix, and maleic anhydride (MAH) coupling agent. The components of the bionanocomposite were parametrized for compatibility with the AMBER14SB force fields. The elastic properties of pure PP systems converge for the chains with at least 20 monomers. The ratio of cellulose in CNF-PP bionanocomposites strongly affects their elastic properties. The elastic modulus of CNF-PP bionanocomposites shows small improvement when the adhesion between hydrophobic and hydrophilic components is facilitated by a MAH coupling agent. The results demonstrate how fully-atomistic MD simulations can be systematically used to evaluate the elastic properties of CNF-PP bionanocomposites and to make predictions that are in agreement with experiments.

## 1. Introduction

Polyolefin-based plastics such as polyethene and polypropylene (PP) are widely used materials in applications such as packaging, logistics containers, and various land and air vehicles. The viscoelastic mechanical properties of polyolefins and their relatively low cost play a key role in their prominence as a ubiquitous material resource. Due to the importance and widespread use of polyolefins, various studies have explored the possibility of creating polyolefin-based composites where the strength and elasticity of the polyolefin are further improved [1,2,3,4,5,6,7].

Natural fibres such as cellulose, hemicellulose, chitin, and lignin are an example that has shown huge potential in the development of polyolefin-based biocomposite materials with improved mechanical properties [8,9,10,11]. Cellulose, being one of the most abundant biopolymers on earth, presents an opportunity to explore the applications of biopolymers in sustainable food packaging materials, medicine, and fuel. Careful selection of cellulose components in biocomposites has exhibited other advantages such as cost-effectiveness, eco-friendliness, and biodegradability [12,13,14]. These characteristics are a major benefit if such biocomposites can be fabricated with physical properties that surpass those of polyolefin-based plastics.

A key challenge repeatedly addressed in PP-biocomposite material development is the opposing polarity of the PP and natural fibre components, resulting in poor adhesion in the final biocomposite [15,16]. The compatibility of the components plays a vital role in the fabrication and properties of the biocomposite material, and it is known that improved adhesion by adding coupling agents between the hydrophilic cellulose fibres and the hydrophobic polymer chains results in improved mechanical properties [17,18,19]. The complex intertwining of polymer chains in the biocomposite matrix and the water-absorbent nature of cellulose fibres further increases the complexity, scalability, and resource requirements for the experimental synthesis of new materials. Molecular modelling techniques with fully-atomistic computational models offer a significantly cheaper and faster approach for the comparative study of the biocomposite structure, component ratios, polymer chain length, and their impact on mechanical properties.

Here, we report a computational approach to generating microscopic models of cellulose nanofibril-polypropylene bionanocomposites (CNF-PP). We evaluate their elastic moduli and Poisson’s ratio using force field-based molecular dynamics (MD) simulations. The individual components of the biocomposite models are validated separately and are in quantitative agreement with the experimental data. We generate the structure models for pure PP unit cells and CNF-PP biocomposite models using simulated annealing (MD) simulations. We implement uniaxial deformation with MD simulations to compute the mechanical properties in the linear regime (bulk modulus, elastic modulus, and Poisson’s ratio). We evaluate the effect of nanocellulose and PP polymer chain lengths and composition ratios on the mechanical properties of the bionanocomposite. We also report ideal unit cell sizes and PP chain lengths to consider when generating computational CNF-PP biocomposite models. Furthermore, we investigate the effects of adding maleic anhydride (MAH) coupling agents to link the hydrophilic (PP) and hydrophobic (CNF) components of the bionanocomposite.

## 2. Methods

### 2.1. Structure Models

**Figure 1:**
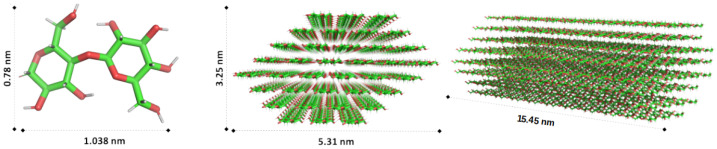
**Left**: Schematic representation of 1–4 linked Iβ cellulose molecular chain (color coding: carbon atoms are green, oxygen atoms are red, and hydrogen atoms are gray). **Center** and **right**: Two different views of a cellulose nanofibril (CNF) with 36 chains and 30 monomers per chain. The models were generated using the Cellulose Builder tool [20].

The structure models of cellulose were implemented in nanofibril form with varying chain lengths. Cellulose Iβ molecular chains are used as the building blocks to construct the initial nanofibril structure (see Figure 1) with the Cellulose Builder package [20]. A nanofibril model comprises 36 chains resembling the cellulose structures forming an insoluble and rigid material around plant cell walls [21]. The structure was modelled with periodic boundary conditions to mimic a macroscopic chain length. Here we consider a nanofibril cellulose model instead of microfibril materials commonly studied in experimental work, as the latter would increase the model size to tens of millions of atoms, making it difficult to model an atomistic picture. However, we believe these atomic-scale studies can lay a foundation to define coarse-grained parameters for cellulose and polypropylene, which can facilitate a mesoscopic model of bionanocomposites.

The Polymer Builder tool available in the CHARMM-GUI interface was used to generate initial structures for isotactic PP with varying chain lengths (3–100 monomers) [22]. The tool generates a matchstick-like untangled geometry for the polymer chain. These are then subjected to simulated annealing, as discussed in the following section.

Bionanocomposite structure models are generated starting with a CNF structure centered in a unit cell, with the fibril parallel to the Cartesian *z*-axis. The unit cell is then enlarged in the *x* and *y*-directions, and the Gromacs tool *insert-molecules* is used to insert multiple PP chains to fill the space around the cellulose fibril. The model is then subjected to simulated annealing, as discussed in the following section.

The structure of maleic anhydride (MAH) was taken from the ChemSpider database and manually linked to individual cellulose and PP molecules to generate a covalently linked bionanocomposite model. The covalent bonds are generated based on the models established in the literature for cellulose-anhydride linkage [23], PP-anhydride linkage [24], and PP-MAH-Cellulose linkage [2,25,26]. The MAH molecule was covalently linked to CNF and PP using the Chemical Builder tool in the PyMol-2.3.0 visualisation package [27].

### 2.2. Force Field Parameters

Force field parameters for the cellulose monomer molecules and PP chain are generated to be compatible with the AMBER14SB force field [28]. For the cellulose monomers, we adapt the partial charges, bonded, and non-bonded parameters available for GLYCAM06 force fields [29]. We note that the GLYCAM06 force fields were developed for describing carbohydrates and lipids, but AMBER14SB has shown reliable validation for polymer MD, allowing us to model PP in addition to cellulose chains. New atom types and dihedral scaling are implemented for the cellulose parameters in AMBER14SB to ensure compatibility and accuracy with the GLYCAM06 description [30,31]. We use the *acpype.py* tool to convert the parameters from AMBER to a Gromacs-compatible format [32]. Additionally, the partial charges were re-derived using the two-stage restricted electrostatic potential (RESP) method [33] to reproduce charge parameterisation that is consistent with the AMBER force field. All quantum-chemical geometry optimisations and electrostatic potential calculations are performed using the HF/6-31G* level of theory with the Gaussian16 program package [34], while the RESP fittings were performed with the AmberTools18 package [31].

Based on the work of Wildman et al. [35] on MD force field parameterisation for polymer molecules, we implement a three-residue model for PP chains to describe the starting, internal, and end chain residues (Figure S1 in Supplementary Material). For PP polymer models, the internal residues are defined with a net zero charge. In contrast, the terminal residues have equal and opposing non-zero charges to obtain an overall neutral charged polymer chain. This approach allows modelling chains with varying lengths without the need to re-parameterise the molecule. The atomic partial charges are derived with the two-stage RESP method for n-alkenes [36]. Bonded interaction values are defined from geometry-optimised structures of the PP chains, and the non-bonded parameters and force constants are adapted from the standard alkene descriptions within the force fields. Additionally, we compute the partial atomic charges for PP models with different chain lengths using the same methods to obtain an ideal convergence value for the net charge on all residues. This approach also validates the ideal lower bound for PP chain length in the force field MD description (Figure S2 in Supplementary Material).

The force field description for the maleic anhydride molecule was adapted based on the parameters derived for furan molecules in AMBER force fields [37]. The covalently linked PP-MAH-CNF moiety was taken as the starting structure and optimised to equilibrate the newly formed bonds. Next, the RESP method was used to extract the atomic partial charges and bonded interactions (bond length, angles, and dihedrals) for the MAH-linked residues while freezing the atomic and residue charges on all adjacent cellulose and (poly-)propylene residues in the linked chain. The rest of the parameters for the MAH molecule were adapted from the pre-validated description available in the AMBER14SB force fields.

### 2.3. Computational Details

During the MD simulations, all short-range dispersive interactions (attraction and repulsion) were described by a Lennard-Jones potential with a cut-off of 1.0 nm; the electrostatic interactions are calculated at each time step using the particle mesh Ewald method [38] with a grid spacing of 0.12 nm. The LINCS algorithm is used to constrain bond lengths in the system [39], while SETTLE [40] is used to constrain the internal degrees of freedom of the TIP3P water molecules for the system with cellulose nanofibril in water. The choice of constraints facilitates the propagation of classical molecular dynamics with a 2 fs time step. All force field simulations are performed with the MD package Gromacs, version 2020.5 [41,42,43,44,45,46,47,48,49].

**Figure 2:**
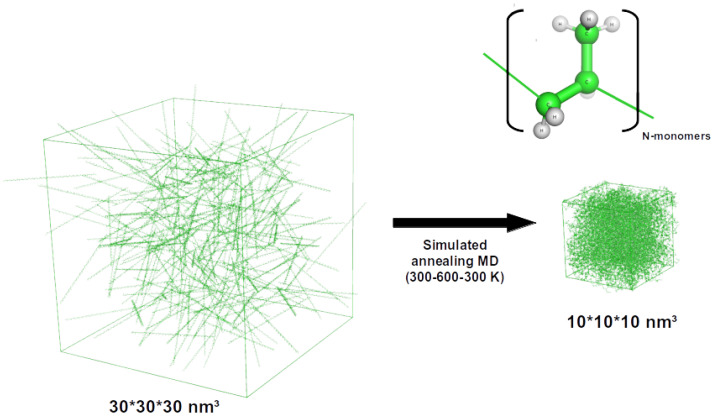
Illustration of the atomistic models used for pure PP systems. The dense PP unit cell on the right is generated using matchstick-like PP chains in a large box (**left**) and running multiple annealing cycles to allow the chains to entangle while achieving a target density of 0.85 g cm${}^{-3}$ (**right**).

Using the PP polymer chains generated using the CHARMM-GUI interface and AMBER14SB parameters, we generate Gromacs-compatible topology files. A cubic unit cell of 10 nm box vector length is defined and filled with the PP chains corresponding to a target density of 0.85 g cm${}^{-3}$. To obtain the ideal PP chain count corresponding to the target density in a chosen unit cell, we compute the total molecular weight of PP chains in a 10 × 10 × 10 nm${}^{3}$ unit cell from the box volume and target PP density. When considering 10-monomer PP chains, a 1000 nm${}^{3}$ cubic box would need to be filled with 1210 chains to obtain 0.85 g cm${}^{-3}$ density. Here, it is crucial to start with a large unit cell (15 × 15 × 15 nm${}^{3}$) to add individual PP chains in the matchstick-like untangled form to avoid any packing bias (Figure 2).

Next, we run a 5000-step Conjugate Gradient (CG) energy minimisation with a 1000 kJ/mol threshold, followed by a 500 ps annealing of the system in NPT ensemble (300 K → 450 K → 300 K) to allow polymer chain entanglement. We observed that the system compresses by a significant volume, resulting in a unit cell with a density of 0.80 g/cm${}^{3}$. The annealing run may crash due to the significant change in box size but can be continued by extracting the last frame or a frame corresponding to the smallest box size or to a density of 0.85 g cm${}^{-3}$. Next, we repeat the annealing MD cycles at higher temperatures to improve the polymer packing by heating the system from 300 to 600 K ten times. Each repeat of 300 → 600 → 300 K occurs over a 500 ps timeframe. If the cell volume and density have not stabilised, the following NPT ensemble run of 50 ns will equilibrate the system to reach the target PP density (0.85 g cm${}^{-3}$) for the system. The equilibration MD run in the NPT ensemble is modelled with the Parrinello–Rahman barostat and 10 ps time scaling to avoid pressure scaling issues.

**Figure 3:**
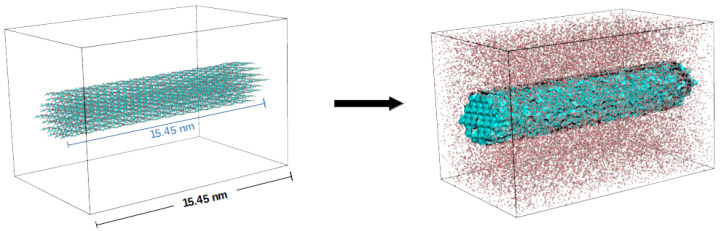
Illustration of the CNF segment in a unit cell before (**left**) and after being solvated in water (**right**). Each molecular chain of the nanofibril comprises 30 cellulose monomers. The unit cell dimensions are chosen to fit a ratio of 30% cellulose by molecular weight. The *z*-axis length is fitted to the fibril length to allow the linking of monomers across the periodic cell, mimicking an infinite fibril.

For simulating the CNF chains with an infinite surface, the terminal glucose monomers on each chain were bonded together across the periodic boundaries of the box. The infinite length model decreases the chain fluctuations and avoids issues introduced with loose terminal segments (Figure 3).

**Figure 4:**
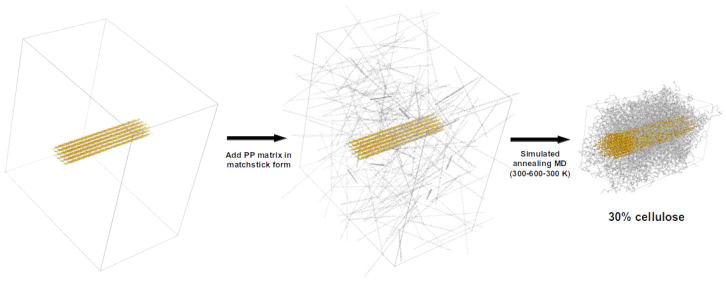
**Left**: Initial structure model for CNF30-PP50 bionanocomposite model (30% cellulose by weight; PP chains with 50 monomers). **Center** and **right**: Modelling steps implemented to generate CNF30-PP50 biocomposite structure models with 30% cellulose by molecular weight. The initial CNF30 and PP50 structures are generated using the Cellulose builder and CHARMM-GUI package, respectively (see text for details).

Using the same protocol implemented for the pure PP model, the CNF-PP biocomposite models are generated with a two-step simulated annealing MD run followed by a 50 ns production MD run in an NPT ensemble (Figure 4). The method allows the generation of a compact cube with a single CNF chain enveloped by PP chains with densities matching the experimental models (0.85 g cm${}^{-3}$). As for the pure CNF model, the terminal cellulose molecule of the CNF chains is linked across the unit cell boundary to replicate the properties of an infinite-length fibril in a smaller unit cell.

### 2.4. Elastic Properties

To study elastic properties, we implement uniaxial compression simulations for all models after the 50 ns equilibration run. The axis deformation protocol is adapted from previous computational studies of the stress–strain relationship for polystyrene and polyimide systems [50,51,52,53]. The deformation mimics the effect of straining the material and allows analysis of the stress response. Here, the uniaxial deformation changes the size of the simulation cell at a constant rate along the positive direction of one of the reference axes, *x*, *y*, or *z* [50]. The isotropic Berendsen barostat [54], with a time constant Δt = 0.5 ps, was replaced with the anisotropic Berendsen barostat with $\tau_{p}$ = 1 ps. In the direction of the applied deformation, the compressibility of the system was set to zero. In the transverse directions, the system compressibility was set to $4.5\times 10^{-10}\;{\mathrm{Pa}}^{-1}$. Therefore, upon stretching, the simulation cell elongates in the direction of deformation and compresses in the directions perpendicular to the deformation in response to the external pressure (1 bar).

During the deformation, the values of the pressure tensor $P_{i},i=x,y,z$, and the simulation cell size $L_{i}$ in the stretching direction were saved every 1 ps. The obtained quantities were converted to the dependence of the stress σ on the relative strain ϵ as

(1) $$\begin{array}{cc} \sigma & =-P_{i}\\ \epsilon & =\left(L_{i}-L_{0i}\right)/L_{0i} \end{array}$$

where $L_{0i}$ is the simulation cell size prior to the deformation (t=0) [55]. The initial part of the dependence σ(ϵ) shows a linear regime of up to about 2% of the deformation ϵ. The elastic modulus *E* is defined as

(2) σ=Eϵ

The value of *E* was determined as the slope of the (linearly approximated) dependence σ(ϵ) in the linear viscoelasticity regime. In some cases, the system had initial residual stress, meaning that the stress–strain dependence does not always start from zero. The error bars in elasticity modulus calculations were computed as mean-square deviations from the average value of *E*, obtained by averaging over three deformation directions, i=x,y,z, and repeating them for three different equilibrated systems.

## 3. Results

All studied systems (CNF in water, pure PP, and CNF-PP biocomposites) were equilibrated for over 50 ns to obtain suitable starting structures for computing mechanical properties. The properties depend on the degree of equilibration, mainly due to unwanted cavity formation when starting from a large simulation cell and using simulated annealing MD simulations to obtain target PP densities of 0.85 g cm${}^{-3}$. The evolution of cell volume and PP densities during the equilibration MD provides validation for such measures (Figure S3 in Supplementary Material).

### 3.1. Effect of the Strain Rate on Elastic Properties

**Figure 5:**
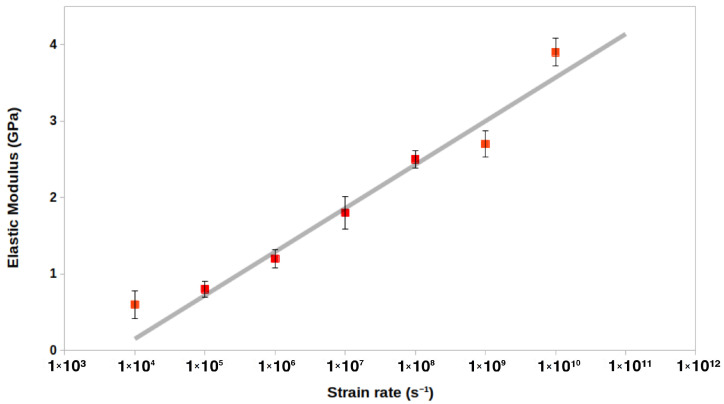
The elastic modulus (4) of the pure PP system with 50 monomers per chain (10 × 10 × 10 nm${}^{3}$ unit cell) shown as a function of the strain rate. Regression fit for strain rates in the range 1.0 × 10${}^{5}$ to 1.0 × 10${}^{8}$ s${}^{-1}$ is shown. The strain rate along the *x*-axis is plotted on a logarithmic scale.

The choice of deformation rate can dictate whether the stress–strain dependence is computed in the equilibrium or non-equilibrium regime. We subjected a model of pure PP with 50 monomer chains to uniaxial strain along all three axes in separate simulations with varying strain rates from 1.0 × 10${}^{4}$ s${}^{-1}$ to 1.0 × 10${}^{10}$ s${}^{-1}$. The fastest strain rate of 1.0 × 10${}^{10}$ s${}^{-1}$ shows significantly higher elastic modulus values as the strain rate of ≈100 m/s likely overestimates the elasticity of the material. The material undergoes significant internal structural changes, similar to the results reported in previous studies on the elastic properties of polymer materials [52,56]. Further, the fastest rates do not allow sufficient time for stress-response through axial relaxation and often lead to brittle fracture. The elasticity values for rates in the range 1.0 × 10${}^{5}$ s${}^{-1}$ to 1.0 × 10${}^{8}$ s${}^{-1}$ agree with a logarithmic fit (Figure 5), which agrees with the previously reported observations from coarse-grained MD for PP and other polymers [52,56,57]. The slowest strain rate of 1.0 × 10${}^{4}$ s${}^{-1}$ does not agree with the fit, as the simulations at such rates start to fall under the equilibrium regime where the system response to stress can average out through untangling and relaxation of the polymer chains, possibly leading to ductile fracture [56]. The ideal choice of strain rate for evaluating elastic properties for PP falls within the logarithmic scale, and here, we use a strain rate of 1.0 × 10${}^{8}$ s${}^{-1}$ for the uniaxial deformation simulations to evaluate the stress-strain relationship with a non-equilibrium process.

### 3.2. Effect of the PP Chain Length on Elastic Properties

**Figure 6:**
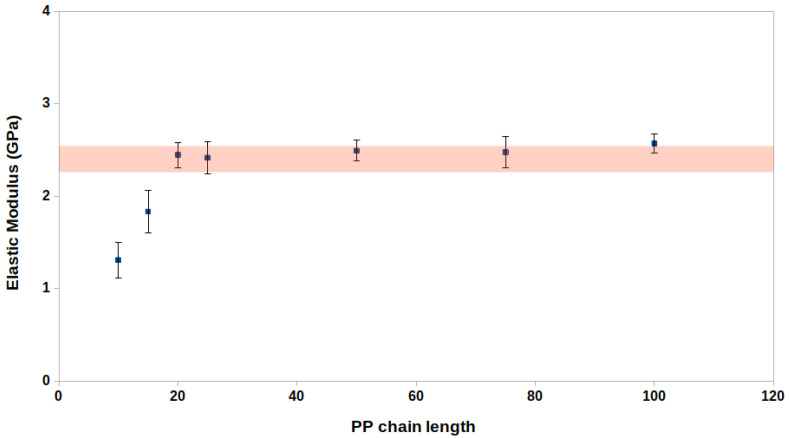
The relationship between PP chain length in terms of monomer units and elastic modulus in the linear regime. The elastic module converges at a chain length of 20 monomer units.

The length of PP chains in real materials can vary from several thousand to hundreds of thousands of monomers. When considering an atomistic picture in simulations, replicating such scales would be computationally too demanding as the systems could take from microseconds to milliseconds to reach a reasonable packing of the PP chains. We model PP chains with lengths varying from 10 to 100 monomers to investigate the influence of the PP chain length on the elastic properties. The elastic modulus was computed for equilibrated models with a simulation cell size of 10×10×10 nm${}^{3}$ and density of ≈0.85 g cm${}^{-3}$. Table 1 and Figure 6 show that the elastic modules converge to approximately 2.3–2.4 GPa for PP systems with a chain length of 20 monomers. The deviations in elastic moduli calculated for chains with 20–100 monomers fall within the error bars for stress–strain relationship calculations. This convergence behaviour is also observed when evaluating the atomic partial charges of the neutrally charged propene residues for force field characterisation (Supplementary Material, Figure S2).

The Poisson’s ratio evaluated from the deformation of axes perpendicular to the direction of strain varies between 0.40 and 0.43 at room temperature (Table 1), closely matching the experimentally known value of 0.38–0.42 [58,59].

Overall, the elastic modulus for pure PP systems is highly dependent on the chain length up to a length of 20 monomers per chain. Considering chain lengths shorter than this may not give a realistic estimate of the elastic properties for PP systems. The choice of PP chain length in atomistic MD simulations is thus essential for computing the elastic properties comparable to experimental data. For the CNF-PP biocomposite models, we model the PP matrix using PP chains with 50 monomers.

### 3.3. Effect of CNF Ratio on Elastic Properties

**Figure 7:**
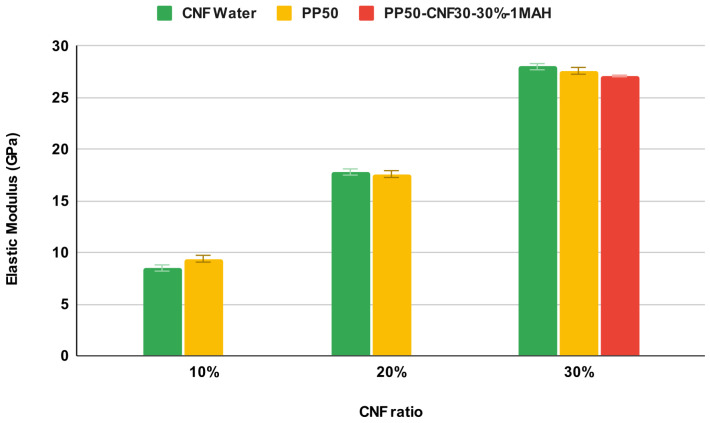
The uniaxial Young’s modulus is determined parallel to cellulose nanofibrils that correspond to 10%, 20%, or 30% of the simulation cell by molecular weight. CNF30 Water is a system where CNF with 30 monomers in a chain is solvated in water. PP50 is a system where CNF30 is embedded in a PP matrix (PP chains with 50 monomers). In PP50-CNF30-30%-1MAH, CNF30 and PP50 are further coupled with a maleic anhydride coupling agent in such a way that the ratio of CNF is 30% by mass percent.

To get an insight into the effect of cellulose nanofibrils on the elastic properties of CNF-PP bionanocomposites, we simulated CNF with 30 monomers in a chain (CNF30) in different types of periodic unit cells with different relative amounts of CNF. We first studied CNF solvated in water. The models were equilibrated similarly to pure PP systems described above, running over 50 ns MD simulations. The cellulose chains were linked across the unit cell along the CNF axis to mimic a macroscopic fibril model (see Figure 3). We studied water-solvated cellulose models with CNF ratios of 10%, 20%, and 30% by molecular weight. We note that considering a periodic model with a CNF ratio beyond 30% can lead to fibril self-interaction due to displacement of the PP matrix. Additionally, under strain, the PP chains would be pushed towards the edge of the cell, creating a model with the cellulose layer sandwiched between PP rather than a fully enveloped CNF model. The elastic modulus in CNF30-water systems is only considered along the direction of CNF chains to exclude the influence of the elastic properties of water. Figure 7 shows that the elastic modulus shows a practically linear correlation to the CNF30 ratio, the elastic modulus being 8.5, 17.8, and 28.0 GPa for CNF30 ratios of 10%, 20%, and 30%, respectively.

**Figure 8:**
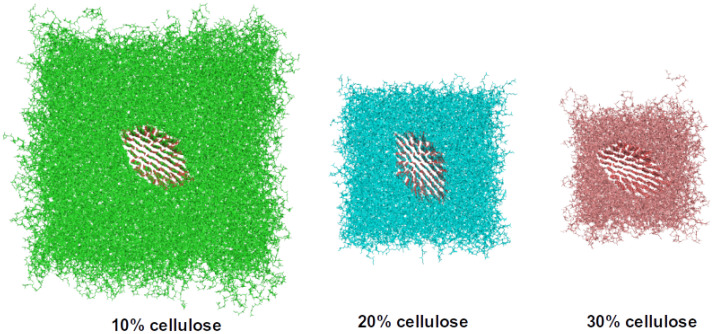
A view in the *xy*-plane on the CNF30-PP50 bionanocomposite models with varying cellulose ratios. Similar CNF30 fibril is in the middle of all models, and the PP chain length is 50 monomers. The *x* and *y*-dimensions of the simulation cells are different in each model, while the *z*-axis length is the same for all three models (approx. 15.5 nm).

The elastic properties were also evaluated for bionancomposite systems with CNF30 embedded in a PP matrix (PP chains with 50 monomers) with varying CNF30 ratios (see Figure 8). All CNF30-PP50 bionanocomposite systems were equilibrated for 50 ns and the density of PP was approximately 0.85 g cm${}^{-3}$, in agreement with the experimental density for PP. The elastic modulus along the CNF is 9.4, 17.6, and 27.6 GPa for CNF ratios of 10%, 20%, and 30%, respectively. The differences in the CNF30-water system are shown as the error bars.

**Figure 9:**
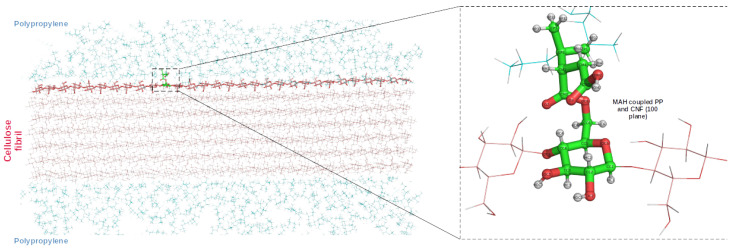
**Left**: A cross-section of the CNF30-PP50-1MAH bionanocomposite. The dashed box highlights the point of covalent linking. **Right**: zoomed-in view of the linking residue formed by the coupling of the internal PP residue (IPP), MAH molecule, and a cellulose residue in the (100) plane.

Finally, we also evaluated the elastic modulus of the CNF30-PP50-1MAH biocomposite model for 30% cellulose by molecular weight to form a base model for the coupling agent-linked bionanocomposite model. Here, the PP and CNF components of the bionanocomposite are covalently linked with maleic anhydride to improve the adhesion (see Figure 9). The elastic modulus along the CNF is 27.1 GPa. The slight decrease compared to CNF30-PP50 can be attributed to the minor distortion on the CNF surface at the site of covalent linking, which breaks the hydrogen bond contact of the linked cellulose chain to its adjacent cellulose chain.

### 3.4. Elastic Properties of CNF-PP Bionanocomposites

**Figure 10:**
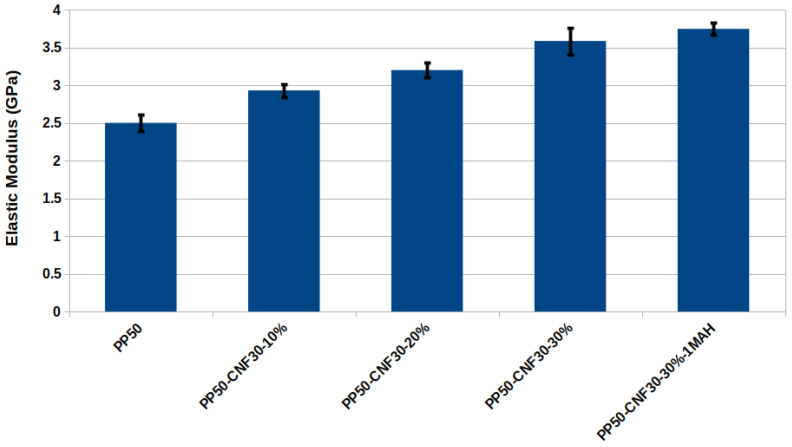
The elastic modulus for pure PP and elastic modulus for several CNF-PP bionanocomposites in the *x* and *y*-directions perpendicular to CNF). The PP density in each model is in the range of 0.83–0.85 g cm${}^{-3}$, which matches the experimental values. The model with the *-1MAH* suffix has a maleic anhydride coupling agent covalently bonded to both PP and CNF.

While the elastic moduli discussed in the previous section were obtained parallel to the infinite cellulose nanofibril, the elastic modulus perpendicular to the CNF (*x* and *y*-directions) gives a more realistic estimate of the macroscopic elastic modulus of CNF-PP bionanocomposites where the CNFs are embedded in a PP matrix. Figure 10 shows a clear correlation between the increase in elastic modulus and CNF ratio in CNF-PP biocomposites, in agreement with experimental observations for natural fibre-reinforced PP composites [59,60,61,62]. The addition of CNF to PP shows an apparent increase in the elastic modulus, which arises from the presence of CNF in the PP matrix. Our results suggest an increase in the PP elasticity by 0.5–1.1 GPa for CNF-PP biocomposites. The biocomposite model with 10% CNF shows an improvement of 20% in the elastic modulus, while the biocomposite with 30% CNF shows an increase of 44%.

When comparing the CNF-PP50-1MAH bionanocomposite model with the MAH coupling agent to CNF-PP50, the material shows a further increase of the elastic modulus to 3.7 GPa. The MAH-coupled system shows an increase of 48% over pure PP and 3% over the CNF30-PP50 model. The slight improvement comes from adding a single MAH molecule as a coupling agent (0.5% by molecular weight). The adhesion between the components through the MAH molecule allows the transfer of stress from PP to stronger cellulose fibres, improving the elasticity of the biocomposite. The measured elastic modulus for the MAH-coupled PP biocomposite is in good agreement with the experimentally evaluated Young’s modulus of 3.45 GPa for a biocomposite system with 30% cellulose [59].

## 4. Conclusions

We have used atomic-scale molecular dynamics simulations to explore the elastic properties of CNF-PP bionanocomposites. The influence of the PP chain length and cellulose ratios on the elastic properties has been evaluated by means of uniaxial deformation simulations. We have investigated microscopic models of CNF, PP, CNF-PP, and CNF-PP-MAH bionanocomposite systems and validated their structural characteristics. The CNF-PP biocomposite models show an increase in elastic modulus compared to pure PP systems by up to 48%. We also found that adding a single MAH molecule to link the CNF and PP only has a small effect on the elastic properties. However, this may change with an increased density of MAH coupling agent molecules. The results illustrate a novel strategy for computational biomaterial design using a systematic computational approach to model cellulose-based bionanocomposite materials and evaluate their elastic properties. The methodology can be efficiently extended to other cellulose-based biocomposites using biodegradable or renewable polymer matrices.

## Figures and Tables

**Table 1:** Elastic properties for pure PP systems with a different number of monomers in the polymer chain. The simulation cell size was 10×10×10 nm${}^{3}$.

PP	Elastic Modulus	Poisson’s Ratio	Density
Monomers	(GPa)		(g/cm${}^{3}$)
10	1.4	0.43	0.83
15	1.9	0.40	0.85
20	2.4	0.40	0.85
25	2.2	0.41	0.84
50	2.5	0.40	0.85
75	2.3	0.40	0.82
100	2.4	0.42	0.82

## Data Availability

Not applicable.

## Supplementary Materials

The following supplementary materials can be downloaded at: https://www.mdpi.com/article/10.3390/nano12193379/s1, Figure S1: Workflow of the molecular dynamics simulations, Figure S2: Stick representation of the three-residue model adapted for PP chains, Figure S3: PP partial charges as a function of PP chain length; Figure S4: Evolution of PP densities during equilibriation runs; Tables S1 and S2: Partial atomic charges generated using RESP methodology.
