# Supplementary material for: Molecular Dynamics Simulations on the Elastic Properties of Polypropylene Bionanocomposite Reinforced with Cellulose Nanofibrils
Source: Nanomaterials (Basel). 2022 Sep 27;12(19):3379. doi: 10.3390/nano12193379 (PMC9565226; doi:10.3390/nano12193379)
Supplement: Supplementary file 1 [file nanomaterials-12-03379-s001.zip › nanomaterials-1924593-supplementary.pdf]

Supporting Information for "Molecular dynamics simulations on the elastic properties of polypropylene bionanocomposite reinforced with cellulose nanofibrils"

Vaibhav Modi and Antti J. Karttunen*

Department of Chemistry and Materials Science,

Aalto University, P.O. Box 16100, FI-00076 Aalto, Finland

* antti.karttunen@aalto.fi

I. SUPPORTING FIGURES

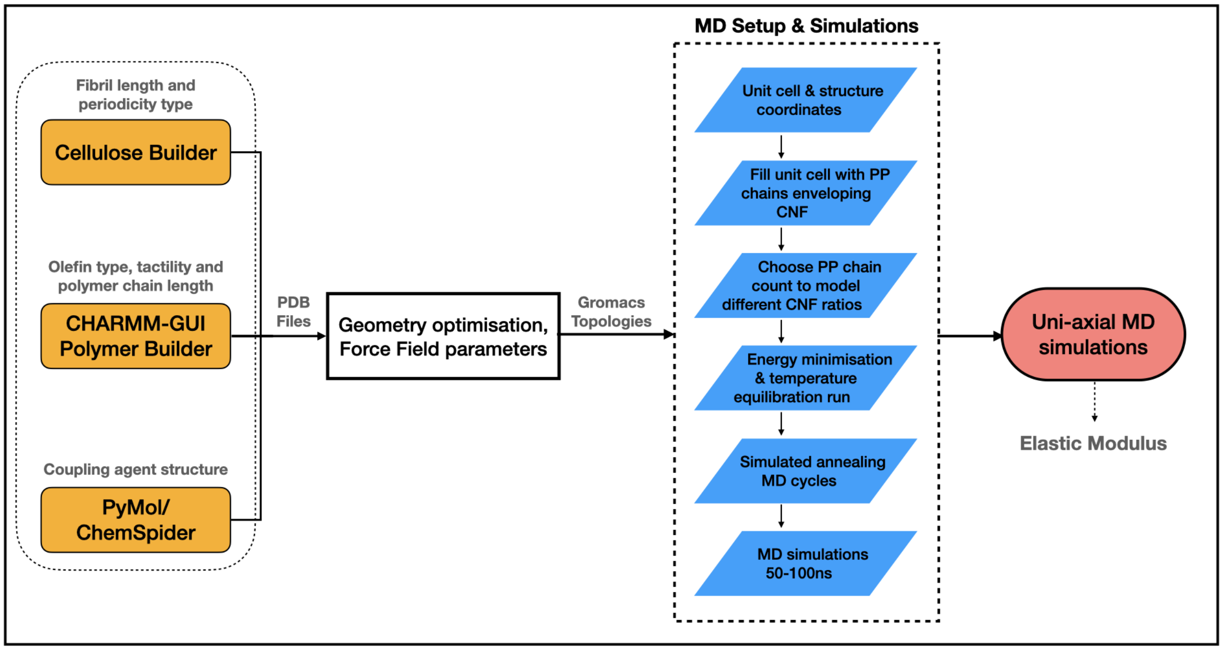

Figure S1. A summary of the workflow to obtain elastic moduli *via* uniaxial molecular dynamics simulations.

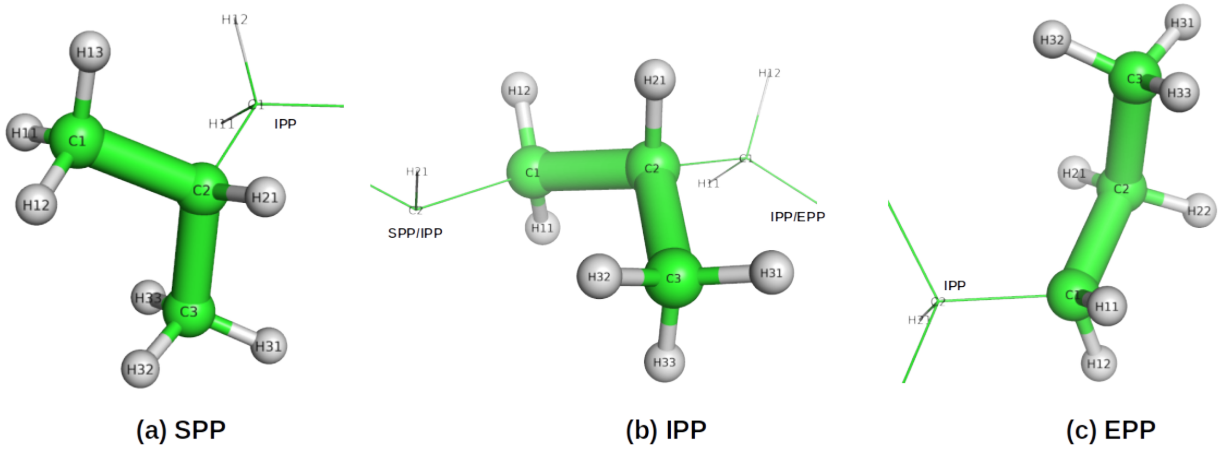

Figure S2. A stick representation of the three-residue model adapted for the polypropylene chains in the force fields description.

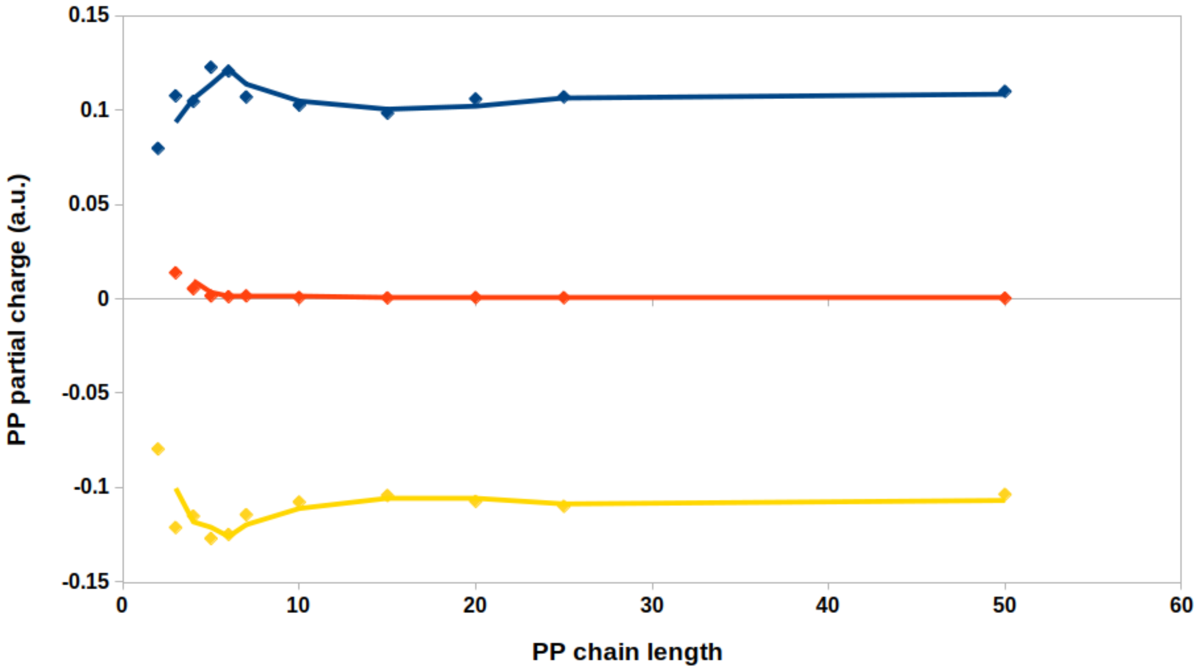

Figure S3. The evolution of partial charge for the starting (SPP), internal (IPP), and ending (EPP) residue of PP chains evaluated using the RESP methodology. The net charge on all three residues converges for PP chains with over 10 monomers and the internal residual charge becomes zero.

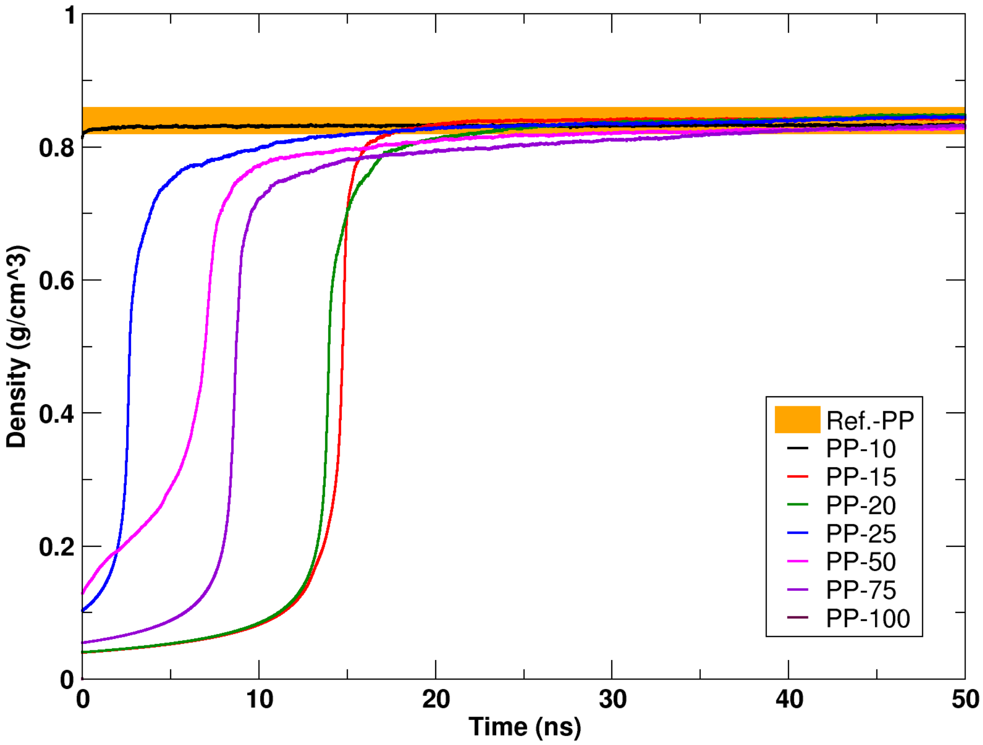

Figure S4. The evolution of PP densities for 50 ns equilibration MD simulations in a $10\times 10\times 10$ nm^3 simulation cell. The target reference density of 0.85 g/cm^3 chosen for pure PP models is shown as an orange line.

II. SUPPORTING TABLES

Table S1. AMBER14SB-compatible atomic partial charges for the polypropylene chain generated using the RESP methodology. A three-residue model was adapted to parameterise the starting (SPP), internal (IPP), and ending (EPP) polypropylene chain residues, which allows using the same parameters to build models of multiple chain lengths. The atom type definitions vary for the C1 and C2 atoms as they form covalent contacts with different number of carbon atoms in the polymer chain (see Figure S2). The values of bonded interactions were defined based on the optimised geometries and the force constants were chosen from the available descriptions of atom types.

Index	Atom name	Atom type	Charges (e^-)		
			SP	IPP	EPP
1	C1	CT/2C/2C	-0.227978	-0.315718	-0.315718
2	H11	HC	0.040948	0.054027	0.054027
3	H12	HC	0.040948	0.054027	0.054027
4	H13	HC	0.040948	-	-
5	C2	3C/3C/2C	0.392106	0.392106	0.388472
6	H21	HC	-0.060022	-0.060022	-0.079459
7	H22	HC	-	-	-0.079459
8	C3	CT	-0.320965	-0.320965	-0.320965
9	H31	HC	0.065515	0.065515	0.065515
10	H32	HC	0.065515	0.065515	0.065515
11	H33	HC	0.065515	0.065515	0.065515
Net Charge (e^-)			0.102530	0.000000	-0.102530

Table S2. Partial atomic charges derived for the linking residue (formed by the coupling the internal PP residue (IPP), MAH molecule, and a cellulose residue). RESP protocol for AMBER14SB compatible parameters was used. Bond lengths and angle parameters were taken from optimised geometries. Dihedral values and force constacts for bonded parameters were defined based on the available values for chosen atom types in the corresponding force fields.

Index	Atom name	Atom type	Charge (e^-)
1	CC1	Cg	0.377123
2	HC1	H2	-0.008252
3	OC5	Os	-0.565086
4	CC5	Cg	0.248547
5	HC5	H1	0.026443
6	CC6	Cg	-0.122684
7	HC61	H1	0.131453
8	HC62	H1	0.131453
9	OC6	OS	-0.35388
10	CC4	Cg	0.050628
11	HC4	H1	0.288059
12	CC3	Cg	0.265514
13	HC3	H1	0.011288
14	OC3	Oh	-0.788778
15	HOC3	Ho	0.524076
16	CC2	Cg	0.177627
17	HC2	H1	0.087939
18	OC2	Oh	-0.755965
19	HOC2	Ho	0.498231
20	OC4	Os	-0.430483
21	CP1	2C	-0.528671
22	HP11	HC	0.151116
23	HP12	HC	0.151116
24	CP2	3C	0.699574
25	CP3	CT	-0.388997
26	HP31	HC	0.078762
27	HP32	HC	0.078762
28	HP33	HC	0.078762
29	CM1	CX	0.030804
30	HM1	H1	-0.006509
31	CM2	C	0.553639
32	O2	O	-0.537653
33	OM2	OH	-0.716363
34	HOM2	HO	0.487029
35	CM3	C	0.895752
36	O3	O	-0.696934
37	CM4	2C	-0.181252
38	HM41	HC	0.028905
39	HM42	HC	0.028905
Net Charge (e^-)			**0.000000**
